# Using Continuing Professional Development with Portfolio in a Pharmaceutics Course

**DOI:** 10.3390/pharmacy4040036

**Published:** 2016-11-07

**Authors:** Jennifer Schneider, Kate O’Hara, Irene Munro

**Affiliations:** School of Biomedical Sciences & Pharmacy, University of Newcastle, Callaghan, NSW 2308, Australia; Kate.Ohara@uon.edu.au (K.O.); Irene.Munro@newcastle.edu.au (I.M.)

**Keywords:** continuing professional development, flipped classroom, critical thinking, simulation, self-directed learning

## Abstract

The introduction of Continuing Professional Development (CPD) to encourage individual life-long learning as a way of maintaining professional competency in pharmacy has faced resistance. To investigate ways to address this barrier we included CPD with portfolio in a university Pharmaceutics course. Underpinning knowledge for the course was delivered using a flipped classroom approach and students used the CPD model to address clinical scenarios presented in a simulated pharmacy setting. Students produced portfolio items for the different case scenarios and submitted these for assessment. This provided the opportunity for students to carry out repeated application of the CPD cycle and, in so doing, develop skills in critical thinking for self-reflection and self-evaluation. This course was designed to encourage the development of higher level learning skills for future self-directed learning. Thirty six students submitted a completed portfolio. Twenty nine students achieved a result of >70%, five students scored between 57%–69%, one student obtained a mark of 50% and one student failed. The end of course survey revealed that while students found portfolio development challenging (40%), they also reported that it was effective for self-learning (54%). Differentiating between the concepts “reflection” and “evaluation” in CPD was problematic for some students and the use of clearer, simpler language should be used to explain these processes in future CPD work.

## 1. Introduction

The fast pace of global research and development has made it increasingly difficult to keep up to date with current knowledge in Pharmacy. With innovations in the development and dispensing of drugs, the delivery of clinical pharmacy services in patient care [1] and the associated acquisition and interpretation of knowledge required for the dissemination of information to patients, it is essential for pharmacists to develop lifelong learning skills [2,3,4].

Continuing education (CE) has been widely used to enable pharmacists to maintain professional competency. It provides for learning through organised experiences and activities [5] and is structured to meet the learning needs of the majority of practitioners [6] but not necessarily the needs of individuals. It has been suggested that it does not include the application of this new learning in the workplace [5], and does not change behaviours or improve practice [7]. To address these shortcomings a different approach to learning is being put in place to enable pharmacists to maintain their competency to practice by encouraging lifelong learning. Continuing Professional Development (CPD) is a systematic ongoing cyclical process of self-directed learning [2,5] that is initiated and driven by the practitioner allowing them to identify their needs, set their learning goals [5], and reflect on their practice [4].

**Figure 1:**
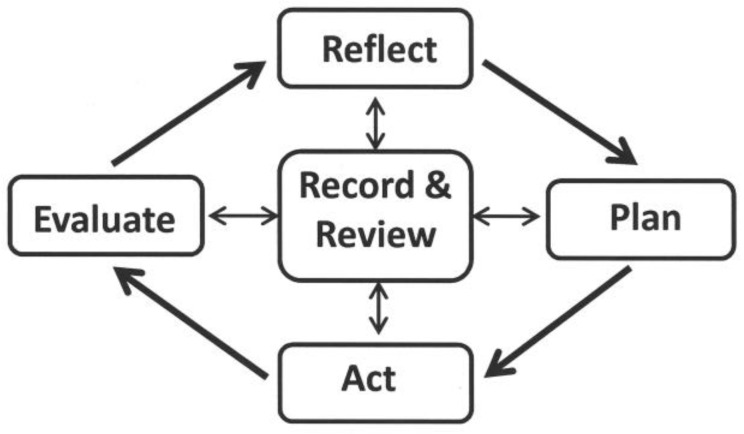
The phases of the Continuing Professional Development (CPD) cycle.

The process of CPD requires the application of higher level learning skills as practitioners reflect on their knowledge and skills and identify their learning needs, develop a plan to address these needs, carry out the plan, and then evaluate and analyse their learning and the value of the activity. Documenting these processes in a portfolio is central to all of these four phases [8,9,10] (Figure 1). As part of the process, practitioners might also be required to synthesise the information they have gathered to create resources for others.

However, the reduction in the organised, expert driven, learning events of CE can be perceived negatively. A qualitative study by Austin et al. (2005) investigating pharmacist’s attitudes, behaviours and preferences for using the CPD model, identified a number of participant concerns. These included concerns about a lack of support from their employer, a lack of role models or mentors, a lack of confidence in their abilities particularly for self-assessment and identifying their personal learning needs, and knowing whether they are doing the right thing [5], and a lack of time. Concerns have also been expressed by pharmacists [11] and other health professionals [9] on the value of recording their development in a learning portfolio. Developing strategies to overcome these potential barriers and identifying ways to encourage and support pharmacists is essential. Austin et al. (2005) suggest that pharmacists may benefit from systematic, structured orientations to CPD and that developing a learning portfolio can address their concerns [11]. It has also been suggested that including professional development skills in university pharmacy programs can provide an opportunity for these students to develop skills for self-assessment [12] and the practice to make self-directed learning a normal part of everyday work.

A university can also provide learning environments, as with the flipped classroom, where students can develop a repertoire of thinking strategies to enable them to acquire, apply, evaluate and synthesize information and knowledge. Learning in a flipped classroom is designed to encourage the development of these higher level learning skills [13] which, according to Miller (2003), comprise the skills needed for critical thinking [14] which is central to CPD. Thus, the aims of this study were (1) to enable our pharmacy students to become lifelong learners through the development of CPD skills with portfolios; and (2) to overcome potential barriers to engaging in CPD through repeated exposure to and familiarisation with the process. The authors have previously used a virtual village pharmacy, The Village Pharm, in a flipped classroom environment for student learning of professional skills in context [15,16], and this appeared to be an appropriate location to simulate scenarios for CPD. This paper describes the introduction of Continuing Professional Development and Portfolio construction at The Village Pharm in a flipped classroom Pharmaceutics course and reports student responses to the experience. The study was approved by the Human Research Ethics Committee at the University of Newcastle, Australia, approval No. H-2013-0151.

## 2. Materials and Methods

CPD with portfolio was an assessable learning experience embedded in Pharmaceutics 3, a Year 1 course offered over 12 weeks in the third trimester of the two year Master of Pharmacy Program at the University of Newcastle, Australia. As part of the flipped classroom approach to learning, a virtual village with a pharmacy and hospital (The Village Pharm) had been developed for course delivery to provide students with the opportunity to experience and apply their learning in context. Four animated case scenarios, created using Crazytalk Pro^®^ (Reallusion Inc., San Jose, CA, USA) and Reallusion Animator Pro^®^ (Reallusion Inc., San Jose, CA, USA) with students assuming the role of pharmacists, were the settings for the CPD experiences. The four scenarios focussed on Medications in Palliative Care, Vaccinations, Pulmonary Drug Delivery Devices, and Diabetes and Blood Glucose Monitoring. 

Using power point students were given a 20–30 min comprehensive overview of the CPD process which began by walking them through the four phases (Figure 1). A sample scenario was established and to start the process the students were asked to list “what do you know” “what do you not know” about the topic. Students were then asked to reflect on their answers and what to do next. In this way the steps involved at each phase were explained and discussed, the inter-connection of the phases established, and examples given to illustrate the type of information to include for the development of the portfolio. Students were provided with recording sheet templates of the four phases to guide their initial portfolio work. They were then introduced to Scenario 1, the first virtual case study which focused on Medications in Palliative Care.

The students assumed the role of pharmacists at the local hospital. Animated characters, Dr. Dorian (Palliative Care Specialist) and Nurse Lochrian (Nurse Manager of the Palliative Care Unit), played out scenes requesting information about different medications for their patients with swallowing difficulties. In other scenarios, the students liaised with the local aged care facility and the community nursing service. To give authenticity to the work, formal letters and medication lists from the doctor and nurse manager were included in the scenarios and formal responses to these requests had to be provided by the students. As a group, the students addressed one phase of the CPD cycle at a time. They were encouraged to discuss the requirements for each phase, ensuring that concepts such as self-reflection, self-appraisal and evaluation were understood. Students then worked individually to address their own needs for each phase and record their work in their portfolios before progressing to the next phase and repeating the procedure. Thus students were guided through the scenario, encouraging consolidation of higher level learning skills (Table 1).

The following second, third and fourth scenarios were presented in a similar way to Scenario 1 during the trimester.

Students were still able to collaborate with their fellow students while they researched the information for the case study but they then worked independently on their portfolios. The completed portfolios were submitted for marking and feedback as part of the course assessment. Students were provided with a marking rubric for their portfolios to indicate the value of components of the work and to guide their approach. Students had to demonstrate correct use of the CPD portfolio structure to reflect the process of building the portfolio, provide full and correct information in response to the questions raised in the different scenarios, and compile and present the requested resources containing clear and appropriate information for professional colleagues and/or patients.

An independent academic, not involved in the teaching of the Pharmaceutics course, observed students working on CPD and portfolio development and then organised voluntary, informal, anonymous discussions with the students to obtain feedback on their experiences. At the end of the Pharmaceutics course students were invited to anonymously complete a survey comprising six open-ended questions about their experiences in the course in general. The survey also included six open-ended questions that were specific to CPD and portfolio development with four of these questions comprising two parts, as shown in Table 2. This provided students with an opportunity to add comments about their experiences. These comments were grouped in to similar themes and the data entered on to a spreadsheet; correct responses were also entered on to a spreadsheet and the results were calculated manually.

## 3. Results

A total of 41 students completed the Pharmaceutics course with 36 students submitting a completed CPD portfolio for marking.

Thirty five of these students were awarded a pass grade or higher for their portfolio work, one student failed and five students chose not submit any work for their portfolio (Table 3).

Only three students presented a portfolio that completely followed the provided CPD structure. A further 13 students presented portfolios with a 4-phase structure but named the first phase as “identify” and the fourth phase as “reflect”. Six students created a 4-phase structure but used different names for the phases and order of presentation. Within these original or re-named and reorganised phases the 22 students correctly presented the information that had been requested in the different scenarios. They also provided the resources requested for patients and/or professional colleagues, with 11 of these students producing very high quality, well designed, interesting and informative materials and another 11 students producing good quality, satisfactorily designed informative materials. Seven students developed a portfolio with a structure of only 3 phases with differing orders of presentation. The omission of a phase resulted in the provision of less information and lower marks. Five students presented the requested information in a number of different structures and two students used no structure at all. Lack of, or modified structures influenced the completeness of the information provided by the students which affected their results (Table 3).

The optional, end of course written survey was completed anonymously by 24 students. The majority of students had correctly completed the phase “reflection” for their portfolios but in the surveys, when asked what this phase involved, only 66.6% (*n* = 16) gave the correct explanation, with 58.4% (*n* = 14) saying that they found it easy and 25% (*n* = 6) saying that it was a difficult process to complete. The students had also correctly completed the phase ‘evaluation’ in their portfolios but only 54.2% (*n* = 13) gave the correct explanation for the process in the survey, with 29.2% (*n* = 7), claiming that the process was easy to complete and 33.3% (*n* = 8) saying that they found it difficult. Results and the number of students who did not respond to these questions is shown in Table 4.

With respect to “reflective self-assessment”, 66.6% (*n* = 16) were able to correctly explain the process, and 33.3% (*n* = 8) found the process easy, while 25% (*n* = 6) thought that it was difficult. In response to the question about the usefulness of recording their work in their portfolio, 66.6% (*n* = 16) reported that it was useful, 25% (*n* = 6) reported that it was not, with 4.2% (*n* = 1) unsure. In response to the question on the effectiveness of CPD and portfolio development as a way to learn, 54.2% (*n* = 13) students reported that it was effective for learning, while 8.3% (*n* = 2) thought that it was just okay. None of the students said that it was not effective. Results and the rate of non-responders to these questions is shown in Table 4.

An open-ended question in the survey asked students what they would like to see changed in the CPD learning exercise if they were given the option (Question 6, Table 2). Responses included making the work less time consuming as they really needed to focus on learning for their exams and others suggested making the work less repetitive.

In the survey, a general question about the Pharmaceutics course asked students which of the learning activities in the course they had struggled with but had really contributed to their learning. For this question the largest proportion of students selected CPD and the learning portfolio as the most difficult but the most helpful for learning, 41.6% (*n* = 10), while 25% (*n* = 6) listed other activities, one student said “none”, and 29.2% (*n* = 7) did not respond. An open question asking students to comment on learning through CPD and maintaining a portfolio attracted 16 responses (66.6%), 15 of which were positive. Comments included that CPD was a good way to learn as it was very effective for self-learning.

The independent observer reported that students appeared to work well together as they progressed through the CPD cycle, collaborating with each other where appropriate. However, a number of international students with English as a second language told the observer that they found it difficult to write about reflection and evaluation as they did not know what words to use as there was no example text to use for word suggestions in their writing.

## 4. Discussion

An aim of this study was to help our students to develop the skills to become lifelong learners by engaging them in CPD activities and the creation of a portfolio to record their work. The CPD learning experience appears to be well placed in the Flipped Classroom where we encouraged the development of higher level learning skills, including applying, evaluating, analysing and synthesising [13], skills that are developed with critical thinking. This is intrinsically linked to CPD, the foundation of which is the application of critical thinking for self-reflection and evaluation. In the application of these skills, the majority of the students proficiently completed the phases “reflect” and “evaluate” in their portfolios although some students named the phase “reflect” as “identify” and the phase “evaluate” as “reflect”. While the information provided in these two phases appropriately addressed “reflect” and “evaluate”, it does indicate some ambiguity in the interpretation of the word headings and what students are being asked to do. The responses to the survey were also confusing in their explanation of “reflection” and “evaluation”, with some students not able to differentiate between the two. Contributing to the confusion is that *reflection*, a fundamental process in CPD, is the point that the pharmacy students *identify* what they need to know. It is also possible that the confusion observed in the explanations expressed by some students might be attributed to their lack of fluency with the English language. It would appear that a greater emphasis needs to be placed on the difference between the processes “reflection” and “evaluation” and the use of clearer, simple language to explain the processes. This will be addressed in future CPD work.

The majority of the students responded positively to CPD and portfolio development as an effective way to learn and this was supported by the excellent grades they received for their portfolio work. Although the students reported that they found portfolio development one of the more challenging activities in the course (40%) they also reported that it was effective in supporting their learning (54%). These results are more positive than the findings reported by Murphy et al. (2011) who investigated the perceived benefits of portfolio development among pharmacy students. They reported that, overall, students thought that portfolio work was not particularly beneficial to their learning with students rating as low the influence of portfolio work on modifying their approach to learning [17]. However, these authors do point out that rating of perceived benefit by students had a large standard deviation, with some student responses claiming the work to be of little value balanced by responses from students who found the work of considerable value. A study by Dolan et al. (2003) involving 219 student nurses also found that 39% of the students felt that their portfolio work did not encourage independent learning [18]. It should also be noted that the cohorts of students surveyed in both studies, *n* = 250 and *n* = 219 respectively were very much larger than in our study. Also the students in the study by Murphy (2011) included students from the first three years of their pharmacy program allowing for a greater range of influences on the students and their work, while our study was limited to just one year of Master of Pharmacy students.

A study by Ashcroft et al. (2006), investigating 154 final year undergraduate pharmacy students’ views on portfolio-based learning, reported that almost two-thirds of the students (63.5%) indicated that portfolio work allowed them to reflect and build on their learning [19]. However, less than half the group (46.7%) said that building a portfolio was a useful learning experience which is considerably lower than the findings in our study. A point of difference between the two studies is that the portfolio work in Ashcroft’s study had a narrower focus and was limited to prescribing while our students had a wider range of experiences in portfolio development. Also, we had a smaller cohort of students involved in our study. Research by Wade and Yarborough (1996) investigating the use of portfolios with 151 student teachers reported that over half of the students (63%) agreed that they learned from the process of reflection in constructing the portfolio [20].These findings are similar to those in our study where more than half (66%) of students reported that recording work in their portfolio was useful and 54% said that it was a helpful way to learn.

Time management [11,21] and a lack of knowledge and confidence [11] are cited as barriers to introducing CPD in the workplace. Thus another aim of this study was to overcome potential barriers to using CPD. We tried to address these issues and build confidence through repeated practice of the process with different scenarios and develop time management skills by setting time limits for completion of the work. However, in the open-ended survey students commented on the repetitive nature of the work and the amount of time it took. Some students also raised these issues with the independent observer. The issue of time was also reported in a study by McMullan (2006) where nursing students felt that portfolio work was very time consuming [22].

As an essential part of the learning process is the application of new knowledge and skills which is more meaningful when it occurs in context, we endeavoured to provide the relevant settings within the simulated Village Pharm. Portfolio development is the agency that supports structured activities to provide for experiential learning and the simulated Village Pharm is the virtual setting. However, the virtual/simulated environment did not completely reflect real-life experiences and some students questioned the relevance/applicability of portfolio development in the work-place. To address this issue in the future, one of the CPD scenarios will be linked to the student placement practice in the course so that, for all students, the experience is real. It is also possible that this change of location will make the CPD work seem less repetitive.

## 5. Conclusions

To address possible barriers to pharmacists engaging in CPD, we explored the suggestion of including professional development skills in a university pharmacy program so that graduates are fluent in the application of higher level learning skills and maintenance of professional competence. Thus, CPD with portfolio was presented largely as a learning tool for the development of skills for future self-directed learning. Students appeared to effectively master the CPD process and their completed portfolios were of a high standard. The low response rate to the survey provided limited information on how students perceived CPD with portfolio work, but it has provided some insight into components that require attention in the future.

## Figures and Tables

**Table 1:** The phases, processes and learning skills in the CPD cycle.

Phase	Process	Active Learning Skills
Reflect	What do you know, what do you have to find out/learn about. (Record your answers on template provided)	Critical thinking, self-appraisal
Plan	Where will you go to find the information; how will it be accessed, gathered, recorded. (Record answers on template provided)	Develop learning objectives
Act	Gather information and expand or summarise as needed. (Record answers on template provided). Formally respond to requests for information. For educational materials, use appropriate understandable language to address needs of professionals or the public.	Synthesise information to create resources
Evaluate	What are the most important things you have learned as a result of these activities? How could your learning assist in future practice as a pharmacist? What was the most difficult part of the process? Do you feel confident you could repeat the process in a different setting? Can this be shared with other colleagues? How? (Record answers on template provided).	Critical thinking: evaluate and analyse effectiveness of learning activities
Feedback: Working with a fellow student, obtain and give feedback on your portfolios and learning materials. This provides an opportunity to see the approach of others		

**Table 2:** CPD questions in the student survey conducted on completion of the course.

1. The first step in the CPD cycle is “reflection”. (a) What are you expected to do at this step? (b) How easy or difficult was this?
2. The fourth step in the CPD cycle is “evaluation”. (a) What are you expected to do at this step? (b) How easy or difficult was this?
3. You recorded the work for your CPD in a portfolio. How useful was this?
4. Did you find CPD with portfolio a helpful way for you to learn?
5. (a) Explain the process of reflective self-assessment. (b) How easy or difficult was this process?
6. (a) If you were to change any part of the CPD learning exercise, what would it be? (b) Why would you make this change?

**Table 3:** Student results for their portfolio work (*n* = 41).

Results %	*n*
≥90	11
80–89	11
70–79	7
57–69	5
50	1
25	1
no submission	5

**Table 4:** Student responses to the end-of-course questionnaire survey (*n* = 24).

Survey Questions	% (*n*)	% (*n*)	% (*n*)	% (*n*)
	Correct	Confused	No response	
In CPD cycle, what does “reflection” involve	66.6 (16)	25 (6)	8.3 (2)	
In CPD cycle, what does “evaluation” involve	54.2 (13)	29.2 (7)	16.6 (4)	
What does “reflective self-assessment” involve	66.6 (16)	0	33.3 (8)	
	Easy	Difficult	No response	
How easy/difficult was the process of “reflection”	58.4 (14)	25 (6)	16.6 (4)	
How easy/difficult was the process of “evaluation”	29.2 (7)	33.3 (8)	37.5 (9)	
How easy/difficult was “reflective self-assessment”	33.3 (8)	25 (6)	41.6 (10)	
	Useful	Not useful	No response	Unsure
How useful was recording work in your portfolio	66.6 (16)	25 (6)	4.2 (1)	4.2 (1)
	Effective	Okay	No response	Unsure
Did you find CPD/portfolio a helpful way to learn	54.2 (13)	8.3 (2)	33.3 (8)	4.2 (1)
